# Clinical Outcome of Hypertrophic Cardiomyopathy in Probands with the Founder Variant c.913_914del in *MYBPC3*: A Slovenian Cohort Study

**DOI:** 10.1007/s12265-024-10551-5

**Published:** 2024-08-19

**Authors:** Nina Vodnjov, Aleš Maver, Nataša Teran, Borut Peterlin, Janez Toplišek, Karin Writzl

**Affiliations:** 1 University Medical Centre Ljubljana, Clinical Institute of Genomic Medicine, Ljubljana, Slovenia; 2 Biotechnical Faculty, University of Ljubljana, Ljubljana, Slovenia; 3 Department of Cardiology, University Medical Centre Ljubljana, Ljubljana, Slovenia; 4 Faculty of Medicine, University of Ljubljana, Ljubljana, Slovenia; 5 European Reference Network for Rare, Low Prevalence, Or Complex Diseases of the Heart (ERN GUARD-Heart), Amsterdam, The Netherlands

**Keywords:** Cardiogenetics, Cardiology, *MYBPC3*, Hypertrophic Cardiomyopathy, HCM, C.913_914del, *MYBPC3: *c.913_914del

## Abstract

Hypertrophic cardiomyopathy is often caused by pathogenic *MYBPC3* variants. The study of Italian patients with HCM and *MYBPC3*(NM_000256.3):c.913_914del showed a higher disease penetrance in males and a higher frequency of arrhythmias compared to patients with other likely pathogenic and pathogenic (LP/P) *MYBPC3* variants. We investigated the clinical outcomes of Slovenian probands with *MYBPC3* LP/P variants, estimated the variant penetrance and compared the results with an Italian study. We identified 31 haplotype-matched individuals with *MYBPC3*:c.913_914del and 34 individuals with other LP/P *MYBPC3* variants. We observed some significant differences in clinical and echocardiographic characteristics and frequency of adverse cardiac events between Slovenian and Italian probands with *MYBPC3*:c913_914del. We were unable to replicate previous findings for *MYBPC3*:c.913_914del, highlighting the complexity of genotype–phenotype associations.

**Graphical Abstract:**

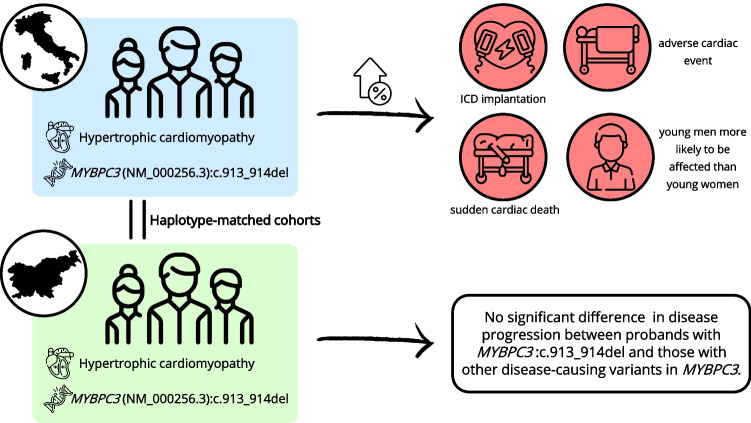

**Supplementary Information:**

The online version contains supplementary material available at 10.1007/s12265-024-10551-5.

## Introduction

Hypertrophic cardiomyopathy (HCM) is considered to be the most common inherited heart disease, with an estimated prevalence of 1:200 [1]. The disease manifests as thickening of the left ventricular (LV) wall of the heart in the absence of any cardiac, syndromic or metabolic cause that could explain the thickening [2]. Individuals with pathogenic HCM-associated genetic variants exhibit incomplete penetrance and variable degrees of disease expression. Some of them die suddenly, while others have a normal life expectancy. A minority of patients progress to the terminal stage of the disease [3].

A pathogenic variant is identified in less than half of individuals with HCM who undergo genetic testing [2]. It is usually found in one of the sarcomeric genes. Roughly 40% of individuals with confirmed HCM have a pathogenic variant identified in *MYBPC3* [4]. *MYBPC3* encodes cardiac myosin-binding protein C (cMyBP-C), which is implicated in sarcomere structure and function [5].

The first genetic variants associated with HCM were described in the 1990s [6]. Accumulated data over time has shown that the mere presence of a pathogenic variant in a patient is not yet sufficient to predict their prognosis for the potential development and severity of heart disease at the level of the individual patient, as it may also be influenced by the additional contribution of non-genetic and co-inheritance of additional genetic factors [3]. However, previous studies have shown that patients with HCM and an identified pathogenic variant have a more severe and earlier clinical course than those without [7, 8]. In addition, the course of disease in patients with a pathogenic variant has been shown to vary depending on the gene in which the variant is found [8, 9]. There are also reports on the clinical presentation of specific variants, such as the study of *MYBPC3*:c.913_914del, which was identified as a founder variant in patients with HCM from the Veneto region of Italy. The study found that the variant is significantly associated with an arrhythmic profile in probands with *MYBPC3*:c.913_914del compared to those with other likely pathogenic and pathogenic (LP/P) *MYBPC3* variants, a higher frequency of adverse cardiac events in adults with *MYBPC3*:c.913_914del compared to those without pathogenic *MYBPC3* variants, and is highly penetrant for HCM in heterozygous males over 20 years of age. The assessment of the variant's pathogenicity was based on a study conducted in a single HCM centre involving 97 probands diagnosed with HCM, of whom 19 probands and 45 of their relatives were found to be heterozygous for *MYBPC3*:c.913_914del [10].

In the present study, we collected clinical data of Slovenian probands with LP/P *MYBPC3* variants and reconstructed the haplotype of Slovenian probands with *MYBPC3*:c.913_914del. We then compared the clinical and echocardiographic characteristics, clinical outcomes and disease penetrance between Slovenian probands with *MYBPC3*:c.913_914del, Slovenian probands with other LP/P *MYBPC3* variants and Italian probands with *MYBPC3*:c.913_914del.

## Methods

In an in-house Mendelian disease registry, next-generation sequencing data were screened for probands with LP/P variants in *MYBPC3*. A total of 41 probands and 24 relatives were identified. Reconstruction of the haplotype surrounding the *MYBPC3*:c.913_914del was done in probands with the variant.

### Selection of Patients

In an in-house Mendelian disease registry of next-generation sequencing data, we identified 134 probands referred for suspected hereditary HCM between 2010 and 2022. Only probands with LP/P *MYBPC3* variant(s) were included in the study. In addition, relatives of probands were recruited for assessment of disease penetrance. Two women with two LP/P *MYBPC3* variants in a compound heterozygous state were excluded from the subsequent analyses.

### Sequencing and Data Analysis

All probands underwent next-generation sequencing (NGS) genetic testing. Probands enrolled in the study were tested as follows. Between January 2010 and 2013, 14 probands underwent cardiomyopathy panel sequencing at an external laboratory (GENDIA—Genetic Diagnostic Network, Antwerp, Belgium). Between January 2014 and July 2019, 27 probands underwent clinical exome sequencing and between July 2019 and December 2022, 93 probands underwent exome sequencing. Sequencing and data analysis were performed at the Clinical Institute of Genomic Medicine, Ljubljana, Slovenia, as previously described [11–13]. The median minimum exome coverage for clinical and exome sequencing was 60x, with > 95% of targets covered at least 10 × sequencing depth.

The presence of the variant in the relatives was confirmed by Sanger sequencing. Briefly, the region containing the variant was amplified using specific set of primers. Sequencing data were analysed using the current version of Geneious software at the time of analysis.

### Variant Classifications

Variants were classified according to ACMG/AMP standards, modified by ACGS recommendations [14, 15].

### Haplotype Estimation

Haplotype reconstruction in the Slovenian probands with *MYBPC3*:c.913_914del was performed by loading the sequencing data into IGV [16] and manually screening the alleles present at the single nucleotide polymorphisms (SNPs) used to determine the “disease” haplotype in the Italian probands with *MYBPC3*:c.913_914del [10]. Due to the retrospective nature of the study, the microsatellites could not be analysed in sufficient detail.

### Clinical Characteristics

Medical history was retrospectively collected from the archive. Imaging characteristics were obtained from the first and last available transthoracic echocardiograms (Supplementary Appendix, Table S1).

HCM was confirmed if the left ventricular wall thickness was ≥ 15 mm in probands and ≥ 13 mm in relatives with a positive genetic test, excluding overload conditions [17]. A positive family history of SCD was defined as the sudden death of a first-degree relative under 40, with or without an HCM diagnosis, or the sudden death of any first-degree relative at any age with an HCM diagnosis [3]. A family history of HCM was considered positive if the relative(s) had clinico-echocardiographically confirmed HCM [10]. Diastolic dysfunction was graded as recommended by clinical guidelines [18]. Left ventricular outflow tract (LVOT) obstruction was defined as a resting or provoked peak gradient ≥ 30 mm Hg [19]. Adverse cardiac events used to calculate event-free survival included successful cardiopulmonary resuscitation/defibrillation for cardiac arrest due to ventricular fibrillation, appropriate ICD shocks, refractory heart failure-related death (NYHA III-IV) and heart transplantation. Where available, HCM SCD risk was determined using data from the proband's last cardiac examination [20].

### Statistical Analysis

Student's t-test for continuous variables and either χ2 test, Fisher exact test or ANOVA for categorical variables were used to detect significant differences between cohorts. The Bonferroni test was used as a post hoc test. Categorical variables are expressed as the number of probands, while the continuous variables are expressed as the mean ± standard deviation, with the range in parentheses. Values with *p* < 0.05 were considered significant.

The probability of experiencing a cardiac event over a lifetime was determined by using the individual's date of birth as the starting point for the time-to-event analysis. Individuals were censored at the time of their first event. The event-free survival rates were calculated using the Kaplan–Meier method, and differences in the event-free survival rates between the cohorts were assessed using the log-rank test.

## Results

### Genetic Analysis

Twenty-two LP/P variants in *MYBPC3* were identified in the Slovenian probands. *MYBPC3*:c.913_914del was identified in 19 probands, *MYBPC3*:c.1484G > A and *MYBPC3*:c.772G > A in two probands and the other 19 variants in only one proband (Supplementary Material, Table S2).

Two women were found to be compound heterozygotes for two LP/P *MYBPC3* variants. One had c.913_914del and c.26-2A > G, and the other had c.25 + 1G > A and c.772G > A. The former presented with clinical signs at the age of 50 and the latter at the age of 13. In addition, one man was a compound heterozygote for one pathogenic and one variant of uncertain significance in *MYBPC3*, c.906-36G > A and c.3142C > T, respectively. He was diagnosed at the age of 35 years. No other LP/P variants were found in other HCM-related genes in the remaining probands.

### Haplotype Analysis in Slovenian Probands with *MYBPC3*:c.913_914del

The sequence surrounding the *MYBPC3*:c.913_914del in Slovenian probands with the variant was screened using the previously reported marker SNPs [10]. In ten (56%) Slovenian probands with *MYBPC3*:c.913_914del the same haplotype as reported in the Italian probands could be reconstructed and in the remaining probands (44%) it differed in only one marker SNP (Table 1). However, this observation supports the hypothesis that both cohorts of probands most likely have a common ancestor.

**Table 1:** Haplotype analysis in Slovenian probands with *MYBPC3*:c.913_914del

reference SNP IDs									
	**rs3729986**	**rs3218719**	**rs11570050**	**rs11570051**	**rs3729989**	**variant**	**rs2856650**	**rs11570078**	**rs2290146**
**ITA haplotype**	**G**	**C**	**delC**	**C**	**A**	**delTT**	**T**	**G**	**C**
**SLO proband 1**	G/G	C/C	delC/delC	C/C	A/A	CTT/delTT	C/C	G/G	C/C
**SLO proband 2**	G/G	C/C	CC/delC	C/C	A/A	CTT/delTT	C/T	G/G	C/T
**SLO proband 3**	G/G	C/C	delC/delC	C/C	A/A	CTT/delTT	C/C	G/G	C/C
**SLO proband 4**	G/A	C/C	delC/delC	C/C	A/A	CTT/delTT	C/T	G/G	C/C
**SLO proband 5**	G/G	C/C	CC/delC	C/T	A/A	CTT/delTT	C/T	G/G	C/T
**SLO proband 6**	G/A	C/C	delC/delC	C/C	A/A	CTT/delTT	C/T	G/G	C/C
**SLO proband 7**	G/G	C/C	delC/delC	C/C	A/A	CTT/delTT	C/C	G/G	C/C
**SLO proband 8**	G/G	C/C	delC/delC	C/C	A/A	CTT/delTT	C/C	G/G	C/C
**SLO proband 9**	G/G	C/C	delC/delC	C/C	A/A	CTT/delTT	C/T	G/G	C/C
**SLO proband 10**	G/G	C/C	CC/delC	C/C	A/G	CTT/delTT	C/T	G/A	C/C
**SLO proband 11**	G/G	C/T	delC/delC	C/C	A/A	CTT/delTT	C/C	G/G	C/C
**SLO proband 12**	G/G	C/C	delC/delC	C/C	A/A	CTT/delTT	C/T	G/G	C/T
**SLO proband 13**	G/G	C/C	delC/delC	C/C	A/A	CTT/delTT	C/T	G/G	C/C
**SLO proband 14**	G/G	C/C	delC/delC	C/C	A/A	CTT/delTT	C/C	G/G	C/C
**SLO proband 15**	G/G	C/C	delC/delC	C/C	A/A	CTT/delTT	C/C	G/G	C/C
**SLO proband 16**	G/G	C/C	delC/delC	C/C	A/A	CTT/delTT	C/C	G/G	C/C
**SLO proband 17**	G/G	C/C	CC/delC	C/C	A/A	CTT/delTT	C/T	G/G	C/T
**SLO proband 18**	G/G	C/C	CC/delC	C/C	A/A	CTT/delTT	C/T	G/G	C/T

*ITA haplotype*, haplotype reported in Italian probands with *MYBPC3*:c.913_914del [10], *SNP ID* single-nucleotide polymorphism identifier (nucleotides at each SNP are reported as allele1/allele2 observed at the locus), *SLO proband* Slovenian proband, dark squares, haplotype observed in Italian probands could not be resembled in a Slovenian proband

### Clinical and Echocardiographic Characteristics of Slovenian Probands with LP/P *MYBPC3* Variants

Clinical and echocardiographic characteristics of the probands with HCM and the LP/P *MYBPC3* variant are shown in Table S1 and compared in Tables 2 and 3, respectively.

**Table 2:** Clinical characteristics of Slovenian probands with *MYBPC3*:c.913_914del (18) and those with other LP/P *MYBPC3* variants (21)

Characteristics	Probands with *MYBPC3:*c.913_914del	Probands with other LP/P* MYBPC3* variants	*p*-value
No. of probands	18	21	NS
**First examination**			
Age [years]	40 ± 17 (1–60)	41 ± 22 (0–74)	NS
NYHA class			
Class I	5	5	NS
Class II	7	10	NS
Class III	1	5	NS
Class IV	0	0	NS
Symptoms			
Dyspnoea	4	12	NS
Chest pain	2	4	NS
Presyncope	2	2	NS
Syncope	1	3	NS
Presence of AF	3	1	NS
Presence of NSVT	0	3	NS
Presence of ICD	0	1	NS
**Last examination**			
Age [years]	52 ± 15 (1,75–68)	52 ± 18 (11–76)	NS
NYHA class			
Class I	3	6	NS
Class II	11	9	NS
Class III	1	4	NS
Class IV	1	0	NS
Symptoms			
Dyspnoea	2	3	NS
Chest pain	3	3	NS
Presyncope	0	1	NS
Syncope	2	3	NS
Presence of AF	4	4	NS
Presence of NSVT	3	5	NS
ICD	3	5	NS

Categorical variables shown as number of probands

*AF* atrial fibrillation, *ICD* implantable cardioverter-defibrillator, *NS* not significant, *NSVT* non-sustained ventricular tachycardia, *NYHA* New York Heart Association

**Table 3:** Echocardiographic characteristics of Slovenian probands with *MYBPC3:*c.913_914del (18) and those with other LP/P *MYBPC3* variants (21)

Characteristics	Probands with *MYBPC3:*c.913_914del	Probands with other LP/P* MYBPC3* variants	*p*-value
No. of probands	18	21	NS
**First examination**			
MLVWT [mm]	20 ± 9 (8–50)	17 ± 4 (8–30)	NS
LV EDVI [mL/m^2^]	58 ± 9 (51–79)	58 ± 11 (36–77)	NS
LAVI [mL/m^2^]	38 ± 11 (23–57)	52 ± 22 (27–100)	NS
LAD [mm]	41 ± 7 (30–55)	43 ± 11 (30–71)	NS
Diastolic dysfunction			
mild	6	6	NS
moderate	6	8	NS
severe	1	1	NS
sPAP [mm Hg]	28 ± 5 (21–37)	32 ± 10 (21–47)	NS
NTproBNP [ng/L]	1 143 ± 1574 (106–4 600)	1 038 ± 867 (85–2 749)	NS
EF [%]	62 ± 9 (47–77)	65 ± 10 (47–77)	NS
Presence of LVOTO	0	4	NS
Presence of LV AA	1	1	NS
**Last examination**			
MLVWT [mm]	18 ± 6 (7–31)	17 ± 4 (9–25)	NS
LV EDVI [mL/m^2^]	68 ± 12 (54–95)	69 ± 13 (45–93)	NS
LAVI [mL/m^2^]	61 ± 30 (27–148)	64 ± 29 (31–155)	NS
LAD [mm]	46 ± 9 (31–66)	48 ± 10 (31–67)	NS
Diastolic dysfunction			
mild	4	5	NS
moderate	8	14	NS
severe	3	1	NS
sPAP [mm Hg]	34 ± 13 (13–59)	33 ± 12 (18–62)	NS
NTproBNP [ng/L]	1 259 ± 829 (261–2 695)	1 352 ± 1 019 (365–4 002)	NS
EF [%]	56 ± 10 (40–76)	59 ± 10 (43–80)	NS
Presence of LVOT obstruction	2	3	NS
Resting LVOT gradient [mm Hg]	8.5 ± 7.0 (3–33)	12.6 ± 22.1(3–100)	NS
Provoked LVOT gradient [mm Hg]	16.4 ± 17.0 (4–60)	17.2 ± 29.1 (4–100)	NS
Presence of LV AA	3	1	NS
HCM Risk SCD Score	3.0 ± 2.0 (1.1–8.1)	3.6 ± 2.7 (1.1–12.0)	NS

Categorical variables shown as number of probands

*AA* apical aneurism, *EDVI* end–diastolic volume index, *EF* ejection fraction, *HCM* hypertrophic cardiomyopathy, *LAD* left atrial diameter, *LAVI* left atrial volume index, *LV* left ventricle, *LVOT* left ventricle outflow, *MLVWT* maximal left ventricle wall thickness, *NA* not applicable, *NS* not significant, *NT-proBNP* N-terminal prohormone of brain natriuretic peptide, *SCD* sudden cardiac death, *sPAP* systolic pulmonary artery pressure

The *MYBPC3*:c.913_914del cohort consisted of 18 probands, 13 (72%) were male. One (6%) of the probands reported a family history of HCM and one (6%) reported a family history of SCD. Probands had their first examination at a mean age of 40 ± 17 years (range 1 to 60 years) and their last examination at a mean age of 52 ± 15 years (range 1.75 to 68 years). The mean duration of follow-up was 10.5 ± 12.2 years. Almost three quarters of the probands (72%) had symptoms at their first examination. None of the patients were in end-stage heart failure during their first examination. However, one of them had progressed to this stage and had undergone heart transplantation (Table 4). At the final examination, probands with *MYBPC3*:c.913_914del showed a slightly thinner left ventricular wall, left ventricular and atrial dilatation (mean LV EDVI, LAVI and LAD increases of 10 mL/m^2^, 23 mL/m^2^, and 5 mm, respectively), greater impairment of diastolic function, reduced EF (mean decrease of 6%), increased sPAP (mean increase of 6 mm Hg) and an increased incidence of LV apical aneurysm, NSVT and ICD implantation (Tables 4 and 5). Five (28%) probands had LV EF < 50%, two (11%) had LVOT gradient > 30 mm Hg, but none had both conditions.

**Table 4:** Comparison of clinical and echocardiographic characteristics between 19 Italian probands with *MYBPC3*:c.913_914del (Group 1), 18 Slovenian probands with *MYBPC3*:c.913_914del (Group 2), and 21 Slovenian probands with other LP/P *MYBPC3* variants (Group 3)

Characteristics	Group 1	Group 2	Group 3	*p*-value
*MYBPC3*:c.913_914del	yes	yes	no	
Slovenian group	no	yes	yes	
No. of probands	19	18	21	NS
Male gender	14	13	11	NS
FH HCM	18	1	1	0.00001*,0.00001**
FH SCD	5	1	2	NS
Age at first exam [years]	36 ± 16	40 ± 17	41 ± 22	NS
Age at last exam [years]	54 ± 14	52 ± 15	52 ± 18	NS
Follow-up period [years]	13 ± 8	11 ± 12	9 ± 8	NS
Presence of AF	4	4	4	NS
Presence of NSVT	12	3	5	0.0069*,0.0088**
ICD	11	3	5	0.0170*,0.0280**
**Symptoms**				
Dyspnoea	11	2	3	0.0045*,0.00388**
Chest pain	6	3	3	NS
Syncope	6	2	3	NS
**Echocardiographic characteristics**				
MLVWT [mm]	23 ± 7	18 ± 6	17 ± 4	0.0281*,0.0048**
LAD [mm]	49 ± 8	46 ± 9	48 ± 10	NS
LV EDVI [mL/m^2^]	65 ± 25	68 ± 12	69 ± 13	NS
EF [%]	54 ± 10	56 ± 10	59 ± 10	NS
Presence of LVOT obstruction	3	2	3	NS
HCM Risk SCD Score [%]	6.3 ± 3.5	3.0 ± 2.0	3.6 ± 2.7	0.0021*,0.0101**
**Events**				
SCD/aSCD	6	1	1	0.04*, 0.03**
HF deaths/transplants	3	3	2	NS

Categorical variables shown as number of probands

*aSCD* aborted sudden cardiac death, *AF* atrial fibrillation, *EDVI* end–diastolic volume index, *EF* ejection fraction, *FH* family history, *HCM* hypertrophic cardiomyopathy, *HF* heart failure, *ICD* implantable cardioverter-defibrillator, *LAD* left atrial diameter, *LV* left ventricle, *LVOT* left ventricle outflow, *MLVWT* maximal left ventricle wall thickness, *NS* not significant, *NSVT* non-sustained ventricular tachycardia, *SCD* sudden cardiac death

^*^significant *p*-value between Group 1 and Group 2

^**^significant *p*-value between Group 1 and Group 3

**Table 5:** Comparison of disease penetrance between 30 Slovenians with *MYBPC3*:c.913_914del, 33 Slovenians with other LP/P *MYBPC3* variants and 64 Italians with *MYBPC3*:c.913_914del

	**Slovenians with *MYBPC3*:c.913_914del**	**Slovenians with other LP/P *MYBPC3* variants**	**Italians with *MYBPC3*:c.913_914del**	***p*-value**
Males and Females				
All with the LP/P variant	30	33	64	
All with HCM	20	23	48	
Penetrance [%]	67	70	75	NS
Males				
All with the LP/P variant	17	16	34	
All with HCM	14	13	31	
Penetrance [%]	82	81	91	NS
Females				
All with the LP/P variant	13	17	30	
All with HCM	7	10	17	
Penetrance [%]	54	59	57	NS
Males vs. Females				
Penetrance (M)/(F)	1.5	1.4	1.6	
*p*-value	NS	NS	0.001	

In a cohort of 21 probands with other LP/P *MYBPC3* variants, 11 (52%) were male. One proband (5%) had a family history of HCM, while two (10%) had a family history of SCD. The mean age at the first examination was 41 ± 22 years (range 0 to 74 years), and at the most recent examination, it was 52 ± 18 years (range 11 to 76 years). The average follow-up period was 9 ± 8 years. A cohort of probands with other LP/P *MYBPC3* variants did not significantly differ in any of the characteristic observed (Table 5). Three (14%) probands had LV EF < 50%, and two (10%) had LVOT gradient > 30 mm Hg, but again none had both conditions.

### Comparison of Clinical and Echocardiographic Characteristics Between Two Slovenian and One Italian Cohort

Comparison of clinical and echocardiographic characteristics at the last follow-up between Italian probands with *MYBPC3*:c.913_914del, Slovenian probands with *MYBPC3*:c.913_914del and Slovenian probands with other LP/P *MYBPC3* variants is shown in Table 4.

**Fig. 1:**
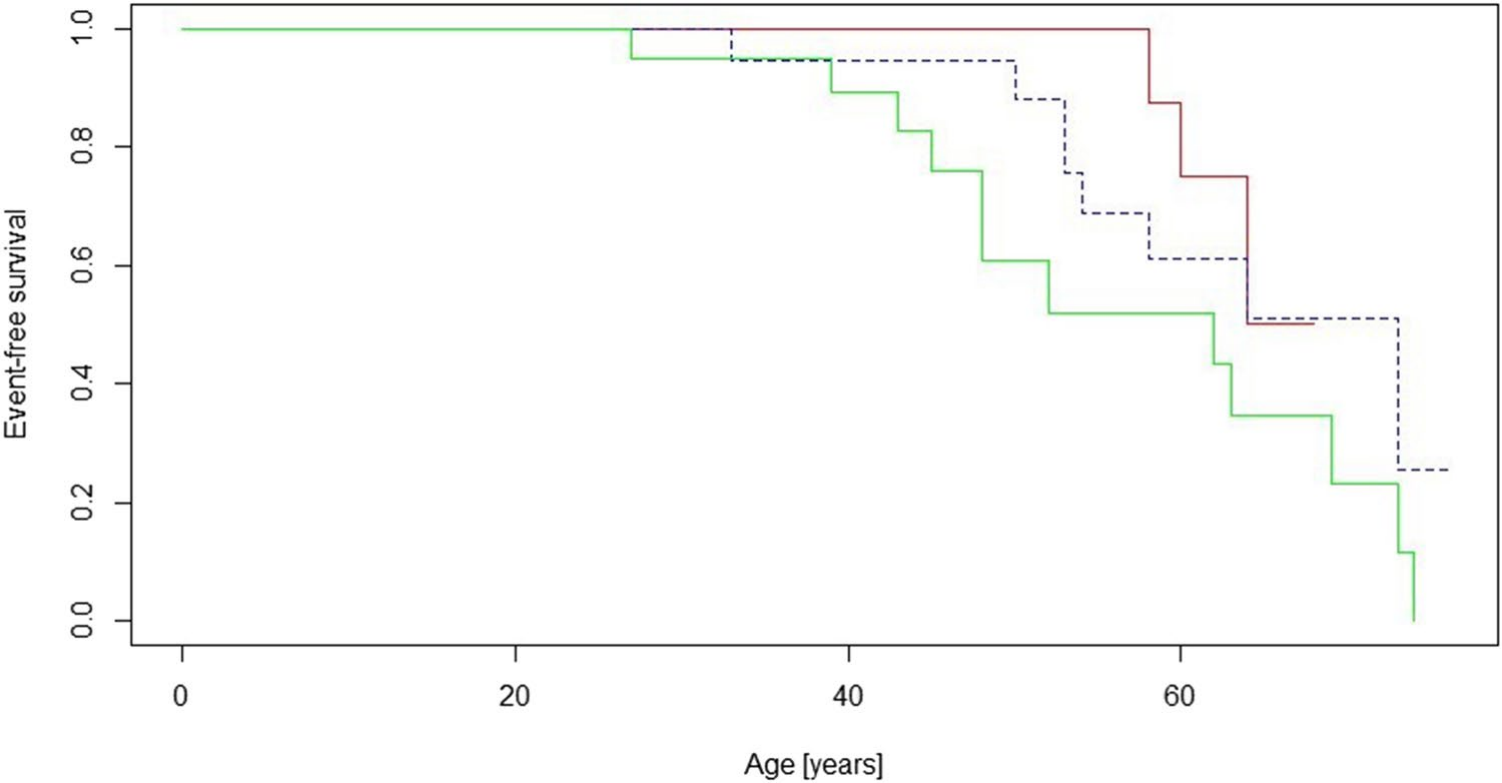
Comparison of long-term survival free of adverse cardiac events between 19 Italian probands with *MYBPC3*:c.913_914del (green), 18 Slovenian probands with *MYBPC3*:c.913_914del (red), and 21 Slovenian probands with other LP/P *MYBPC3* variants (blue)

Italian probands with *MYBPC3*:c.913_914del differ significantly in some aspects from Slovenian probands with *MYBPC3*:c.913_914del and Slovenian probands with other LP/P *MYBPC3* variants, although they were diagnosed and followed up on average at the same age. The most significant difference was the proportion of probands with a family history of HCM, which was strikingly higher in the Italian cohort than in the Slovenian cohorts (95% vs. 6% and 4%) (*p* = 0.00001). On average, the Slovenian probands had a lower incidence of NSVT (63% vs. 17% and 24%, *p* = 0.005), ICD implantations (58% vs. 17% and 24%, *p* = 0.016), experienced less dyspnoea (58% vs 11% and 14%, *p* = 0.001), had a lower maximum left ventricular wall thickness (23 ± 7 mm vs. 18 ± 6 mm and 17 ± 4 mm, *p* = 0.004) and had a better prognosis in terms of the HCM Risk SCD prediction score (6.3 ± 3.5 vs. 3.0 ± 2.0 and 3.6 ± 2.7, *p* = 0.001). The only statistical difference in events observed was the frequency of adverse cardiac events (32% vs 6% and 5%, *p* = 0.02) (Table 4). Long-term survival free of adverse cardiac events between the groups is compared in Fig. 1. No statistical difference was observed between the cohorts studied (*p* = 0.09) (Fig. 1).

### Segregation Analysis in Slovenians with LP/P *MYBPC3* Variants

The *MYBPC3*:c.913_914del segregated in 12 of 18 tested relatives, two of whom had the HCM phenotype. Segregation analysis was performed in nine families, three of which included the proband's parents. In all families where the parents were tested, segregation of the variant from the parents to the proband was confirmed, even though the parents reported no heart problems. The two affected relatives with *MYBPC3*:c.913_914del were found to have a mild form of HCM and neither reported any HCM-related symptoms. Other LP/P *MYBPC3* variants segregated in 12 out of 25 relatives tested, two of whom had the HCM phenotype. For more information on the clinical presentation in relatives, see Supporting information.

### Comparison of the Disease Penetrance Between Two Slovenian and One Italian Cohort

The overall and gender-specific disease penetrance did not differ significantly between the cohorts (Table 5). Gender penetrance did not differ significantly in cohorts of Slovenians with *MYBPC3*:c.913_914del and Slovenians with other LP/P *MYBPC3* variants, but it did in a cohort of Italians with *MYBPC3*:c.913_914del (*p* = 0.01).

## Discussion

The present study characterised Slovenian probands with LP/P *MYBPC3* variants clinically and echocardiographically and compared characteristics, clinical outcomes and estimated disease penetrance between Slovenian probands with LP/P *MYBPC3* variants and Italians with *MYBPC3*:c.913_914del.

Likely pathogenic and pathogenic variants in *MYBPC3* were identified in 41 (60%) of Slovenian probands with an identified genetic cause of HCM. The most frequently identified variant was *MYBPC3*:c.913_914del, which was present in 19 (46%) individuals with the LP/P *MYBPC3* variant. To date, *MYBPC3*:c.913_914del has been extensively described in Italian patients with HCM. The variant has also been reported in individuals living in the Netherlands, Australia, Canada, USA, Brazil, Belgium, Finland and Germany in ClinVar (variation ID:42,801) [21, 22] and in patients with HCM in the literature [7, 23–27].

The haplotype analysis showed that the probands with *MYBPC3*:c.913_914del from the Italian and Slovenian cohorts most likely share the same common ancestor. The segregation analysis supports this finding, as in both cohorts the variant was found to be inherited from the tested parents (three segregations in the Slovenian cohort and four in the Italian cohort). Considering that Slovenian and Italian probands with *MYBPC3*:c.913_914del share not only a pathogenic variant but also a fairly similar environment, we expected that the findings regarding the penetrance and clinical outcome observed in the Italian cohort would be replicated in the Slovenian cohort. However, the results of the present study did not confirm previous findings.

First, the study of Italian patients with HCM found that the variant was significantly associated with an arrhythmic profile leading to more frequent episodes of NSVT, ICD implantation and a higher risk of SCD, and with a milder thickening of the left ventricular wall in probands with *MYBPC3*:c.913_914del compared to those with other LP/P *MYBPC3* variants [10]. We observed that the Slovenian probands with *MYBPC3*:c.913_914del and those with other LP/P *MYBPC3* variants did not differ significantly in any of the above parameters, but both Slovenian groups had a significantly lower incidence of NSVT, ICD implantation, lower calculated risk of SCD and milder wall thickening than the Italian probands with *MYBPC3*:c.913_914del. Although the results of the present study cannot explain why Italian probands with *MYBPC3*:c.913_914del differ significantly from the Slovenian probands, the comparison between Slovenian probands with *MYBPC3*:c.913_914del and those with other LP/P *MYBPC3* variants may suggest that the clinical presentation of LP/P *MYBPC3* variants is not as different on a larger scale as might be expected. Research to elucidate the effect of specific HCM-causing variants has been ongoing for several decades. It remains unclear why LP/P variants in different genes related to HCM can result in a similar phenotype in unrelated individuals [28] while the same LP/P variant can lead to markedly different phenotypes among family members [8, 29]. To date, there is no evidence that pathogenic *MYBPC3* variants lead to variant-specific phenotypes that have been confirmed by functional studies. Currently, only research using a meta-analysis approach can identify clinically important differences at the gene or variant level [3, 8, 30].

Second, Italian probands with HCM and *MYBPC3*:c.913_914del were found to have a higher incidence of adverse cardiac events compared to those without the pathogenic *MYBPC3* variant. The comparison of the frequency of events between Slovenian probands with the LP/P *MYBPC3* variant and Italian probands with *MYBPC3*:c.913_914del showed that the only statistically significant difference was the frequency of adverse cardiac events between Slovenian and Italian probands with *MYBPC3*:c.913_914del, which was lower in Slovenian probands. However, a recent meta-analysis found no difference in adverse events between patients with HCM with known and unknown genetic background of the disease, suggesting that the effect of the pathogenic variant on the frequency of adverse cardiac events should be studied on a larger scale [8].

Third, the Italian study found that probands with *MYBPC3*:c.913_914del almost always have a family history of HCM (95%) and that the penetrance of the disease for *MYBPC3*:c.913_914del is not only age-related but also gender-specific, as men over 20 years of age with *MYBPC3*:c.913_914del had a significantly higher incidence of HCM than women of the same age. These conclusions were drawn from the segregation analysis, which was performed in 14 out of 19 Italian families. The high proportion of family history for HCM is mainly based on the examination of relatives of the same or younger age as the proband, as only in four families (29%) the inheritance of the variant in the proband was confirmed by the affected parent and in half of the families the clinically unaffected parents were not included in the segregation analysis (5 families, 36%) or information on the clinical status of the parents was not provided (3 families, 21%). This suggests that not only the family history but also the penetrance of the variant was inflared. No significant difference in the overall penetrance of the LP/P *MYBPC3* variants was found in either the Italian or the Slovenian cohorts. We did not observe a significant gender difference in the disease penetrance between Slovenian probands with *MYBPC3*:c.913_914del and those with other L/P *MYBPC3* variants employing the same method as in the Italian study. Limited sample size resulting in insufficient statistical power to detect a statistically significant difference is a possible explanation for this finding, given the known male predominance in inherited cardiomyopathy cohorts [3, 8, 30]. Another possible explanation lies in the limitations of the traditional family history-based method for estimating penetrance. This approach may provide information that is valid only for the families studied and may be biased when generalised to the wider population and other world populations. Therefore, especially with the increasing availability of data, population-based studies of unrelated individuals with pathogenic variants provide a more reliable method for estimating disease penetrance [30, 31].

Our findings in the Slovenian cohort with the *MYBPC3*:c.913_914del variant differed from those reported in the Italian study, suggesting that conclusions about the clinical presentation of a specific pathogenic variant should be drawn with caution, given the incomplete penetrance and heterogeneous clinical presentation of variants associated with hypertrophic cardiomyopathy.

### Study Limitations

In this retrospective study, participants were recruited on the basis of genetic analysis and cascade screening, with clinical and imaging data collected over 20 years of routine medical care, not as part of a targeted clinical trial. Due to advances in cardiology practice over time, some probands had inadequate initial examination data, which may have biased the assessment of changes in echocardiographic parameters over time. Limited cardiac magnetic resonance imaging data prevented an assessment of differences in cardiac fibrosis between the cohorts. However, all probands were followed up, and data from the last examination are complete. Due to limited imaging data from relatives with HCM, only probands were evaluated for the cardiac phenotype. Extensive segregation analysis was not possible for all probands due to logistical constraints.

## Supplementary Information

Supplementary Material 1: Clinical presentation in relatives

Supplementary Material 2: The patient's history from the first and most recent transthoracic echocardiography reports and summary statistics

Supplementary Material 3: Other LP/P *MYBPC3* variants.

## Data Availability

All data generated or analysed during this study are included in this published article and its supplementary information files.

## Abbreviations

AA: Apical aneurism
AF: Atrial fibrillation
cMyBP-C: Cardiac myosin-binding protein C
EF: Ejection fraction
FH: Family history
HCM: Hypertrophic cardiomyopathy
ICD: Implantable cardioverter-defibrillator
ITA haplotype: Haplotype reported by Calore et al. (2015) [10]
LAD: Left atrial diameter
LAVI: Left atrial volume index
LP: Likely pathogenic
LV: Left ventricle
LVOT: Left ventricle outflow tract
MLVWT: Maximal left ventricle wall thickness
NA: Not applicable
NGS: Next-generation sequencing
NS: Not significant
NSVT: Non-sustained ventricular tachycardia
NT-proBNP: N-terminal prohormone of brain natriuretic peptide
NYHA: New York Heart Association
P: Pathogenic
SCD: Sudden cardiac death
SNP ID: Single-nucleotide polymorphism identifier
sPAP: Systolic pulmonary artery pressure
