# Supplementary material for: Clinical Outcome of Hypertrophic Cardiomyopathy in Probands with the Founder Variant c.913_914del in MYBPC3: A Slovenian Cohort Study
Source: J Cardiovasc Transl Res. 2024 Aug 19;18(1):110–20. doi: 10.1007/s12265-024-10551-5 (PMC11885317; doi:10.1007/s12265-024-10551-5)
Supplement: Supplementary file 1 — Supplementary Material 1: Clinical presentation in relatives [file 12265_2024_10551_MOESM1_ESM.docx]

**Clinical characteristics of Slovenians relatives with LP/P *MYBPC3* variants**

The *MYBPC3*:c.913_914del segregated in 12 of 18 tested relatives, two of whom had the HCM phenotype. Segregation analysis was performed in nine families. In three families, *MYBPC3*:c.913_914del was confirmed to have been inherited from one of the parents, none of whom reported any heart problems. Two also underwent echocardiography at the ages of 42 and 62, which showed no cardiac abnormalities. Other probands in whom the origin of the variant was unknown reported that their parents did not have hypertrophic cardiomyopathy, did not have heart problems before the age of 50, or died unexpectedly before the age of 50, except for one proband who reported that the parent died suddenly at the age of 37 with no known underlying heart disease.

The two affected relatives with *MYBPC3*:c.913_914del were found to have a mild form of HCM and neither reported any HCM-related symptoms. One had obstructive HCM and other cardiac comorbidities at the age of 61. The other had non-obstructive HCM, a maximum left ventricular wall thickness of 12 mm, a slightly enlarged left atrium and a bicuspid aortic valve at the age of 42 years.

Other LP/P *MYBPC3* variants segregated in 12 out of 25 relatives tested, two of whom had the HCM phenotype. For one affected relative, the only information available was that the individual had been diagnosed with HCM. The other reported having had heart problems since childhood, but had only been seen in a cardiology clinic since his sixth decade. He was found to have septal thickening of up to 15 mm and arrhythmias, and was implanted with an ICD at the age of 72.
