# Supplementary material for: Clinical Outcome of Hypertrophic Cardiomyopathy in Probands with the Founder Variant c.913_914del in MYBPC3: A Slovenian Cohort Study
Source: J Cardiovasc Transl Res. 2024 Aug 19;18(1):110–20. doi: 10.1007/s12265-024-10551-5 (PMC11885317; doi:10.1007/s12265-024-10551-5)
Supplement: Supplementary file 3 — Supplementary Material 3: Other LP/P MYBPC3 variants. [file 12265_2024_10551_MOESM3_ESM.docx]

Table S2. Other disease-causing *MYBPC3*(NM_000256.3) variants.

| **Other disease-causing *MYBPC3* variants** | **Type of the variant** |
| --- | --- |
| c.1484G>A * | missense |
| c.772G>A * | missense |
| c.1156_1171dup | nonsense |
| c.1351+2T>C | splice |
| c.1467del | frameshift |
| c.1483C>T | missense |
| c.1806del | frameshift |
| c.2373dup | frameshift |
| c.25+1G>A | splice |
| c.2526C>G | nonsense |
| c.26-2A>G | splice |
| c.3190+5G>A | splice |
| c.3233G>A | nonsense |
| c.3490+1G>A | splice |
| c.3627+1G>A | splice |
| c.3767_3769del | in-frame deletion |
| c.3811C>T | nonsense |
| c.3814+1G>A | splice |
| c.772+1G>A | splice |
| c.821+1G>A | splice |
| c.906-36G>A | intron |

* Variants identified in two probands.
